# A uniqueness method to a new Hadamard fractional differential system with four-point boundary conditions

**DOI:** 10.1186/s13660-018-1801-0

**Published:** 2018-08-10

**Authors:** Chengbo Zhai, Weixuan Wang, Hongyu Li

**Affiliations:** 1 School of Mathematical Sciences, Shanxi University, Taiyuan, China; 2 College of Mathematics and Systems Science, Shandong University of Science and Technology, Qingdao, China

**Keywords:** 34A08, 34B27, 34B15, Hadamard fractional derivative, Existence and uniqueness, *φ*-$(h,r)$-concave operator

## Abstract

In this article, we discuss a new Hadamard fractional differential system with four-point boundary conditions

$$\textstyle\begin{cases} {}^{H} D^{\alpha}u(t)+f(t,v(t))=l_{f},\quad t\in(1,e),\\ {}^{H} D^{\beta}v(t)+g(t,u(t))=l_{g},\quad t\in(1,e),\\ u^{(j)}(1)=v^{(j)}(1)=0, \quad 0\leq j\leq n-2,\\ u(e)=av(\xi),\qquad v(e)=bu(\eta),\quad \xi, \eta\in(1,e), \end{cases} $$

where $a,b$ are two parameters with $0< ab(\log\eta)^{\alpha-1}(\log\xi )^{\beta-1}<1$, $\alpha, \beta\in(n-1,n]$ are two real numbers and $n\geq3$, $f,g\in C([1,e]\times(-\infty,+\infty),(-\infty,+\infty))$, $l_{f}, l_{g}>0$ are constants, and ${}^{H} D^{\alpha}, {}^{H} D^{\beta}$ are the Hadamard fractional derivatives of fractional order. Based upon a fixed point theorem of increasing *φ*-$(h,r)$-concave operators, we establish the existence and uniqueness of solutions for the problem dependent on two constants $l_{f}, l_{g}$.

## Introduction

In this article, we discuss the following new Hadamard fractional differential system with four-point boundary conditions:

1.1 $$\begin{aligned} \textstyle\begin{cases} {}^{H} D^{\alpha}u(t)+f(t,v(t))=l_{f},\quad t\in(1,e),\\ {}^{H} D^{\beta}v(t)+g(t,u(t))=l_{g},\quad t\in(1,e),\\ u^{(j)}(1)=v^{(j)}(1)=0,\quad 0\leq j\leq n-2,\\ u(e)=av(\xi),\qquad v(e)=bu(\eta),\quad \xi, \eta\in(1,e), \end{cases}\displaystyle \end{aligned}$$

where $a,b$ are two parameters with $0< ab(\log\eta)^{\alpha-1}(\log\xi )^{\beta-1}<1$, $\alpha, \beta\in(n-1,n]$ are two real numbers and $n\geq3$, $f, g\in C([1,e]\times(-\infty,+\infty), (-\infty,+\infty))$, $l_{f}, l_{g}$ are constants, and ${}^{H} D^{\alpha}, {}^{H} D^{\beta}$ are the Hadamard fractional derivatives of fractional order. A pair of functions $(u,v)\in C[1,e]\times C[1,e]$ is called a solution of system (1.1) if it satisfies (1.1). We will consider system (1.1) under the case $l_{f}, l_{g}>0$. We use a recent fixed point theorem for *φ*-$(h,r)$-concave operators to study system (1.1).

The study of fractional differential equations has made fast development and it has many applications in some fields such as physics, chemistry, engineering, and biological science; see [1–18] and the references therein. We can see that the topic of the work are most Riemann–Liouville and Caputo-type fractional equations. As we know, there is another kind of fractional derivative which can be seen in the literature due to Hadamard introduced in 1892 (see [19]). This kind of derivative includes a logarithmic function of arbitrary exponent in the kernel of the integral appearing in its definition. Recently, there have been some papers reported on boundary value problems of Hadamard fractional differential equations, see [20–34]. Ahmad and Ntouyas [20, 21] discussed some fractional integral boundary value problems involving Hadamard fractional differential equations/systems and obtained the existence and uniqueness of solutions by applying the Banach fixed point theorem and Leray–Schauder alternative, respectively.

In [22], the authors studied the boundary value problem of Hadamard fractional differential inclusions

1.2 $$\begin{aligned} \textstyle\begin{cases} {}^{H} D^{\alpha}x(t)\in F(t,x(t)),\quad 1< t< e, 1< \alpha\leq 2,\\ x(1)=0,\qquad x(e)={}^{H} I^{\beta}x(\eta),\quad 1< \eta< e, \end{cases}\displaystyle \end{aligned}$$

where $F:[1,e]\times(-\infty,+\infty)\rightarrow\varrho(-\infty ,+\infty)$ is a multivalued map, $\varrho(-\infty,+\infty)$ is the family of all nonempty subsets of $(-\infty,+\infty)$. By using standard fixed point theorems for multivalued maps, the existence of solutions was established.

In [24], the authors applied Leggett–Williams and Guo–Krasnoselskii’s fixed point theorems to get multiple positive solutions for Hadamard fractional differential equations on the infinite interval

1.3 $$\begin{aligned} \textstyle\begin{cases}{}^{H} D^{\alpha}u(t)+a(t)f(u(t))=0,\quad 1< \alpha\leq2, t\in (1,\infty),\\ u(1)=0,\qquad D^{\alpha-1}u(\infty)=\sum_{i=1}^{m}\lambda_{i} {}^{H} I^{\beta_{i}}u(\eta), \end{cases}\displaystyle \end{aligned}$$

where $\eta\in(1,\infty), \lambda_{i}\geq0, \beta_{i}>0\ (i=1,2,\ldots,m)$ are constants.

In [35], the author considered positive solutions for the Hadamard fractional differential system

1.4 $$\begin{aligned} \textstyle\begin{cases} {}^{H} D^{\alpha}u(t)+\lambda f(t,u(t),v(t))=0,\quad t\in (1,e),\\ {}^{H} D^{\beta}v(t)+\lambda g(t,u(t),v(t))=0,\quad t\in(1,e),\\ u^{(j)}(1)=v^{(j)}(1)=0,\quad 0\leq j\leq n-2,\\ u(e)=av(\xi), \qquad v(e)=bu(\eta),\quad \xi,\eta\in(1,e), \end{cases}\displaystyle \end{aligned}$$

where $\lambda,a,b$ are three parameters, $\alpha,\beta\in(n-1,n]$ are two real numbers, and $n\geq3$. By applying Guo–Krasnoselskii’s fixed point theorem, at least one positive solution was given.

From the papers mentioned above, we can see that system (1.1) is different from (1.2)–(1.4), and it is a new type of Hadamard fractional differential equations. Motivated by the recent papers [34, 36], we study the uniqueness of solutions for Hadamard fractional differential system (1.1). By using a fixed point theorem of increasing *φ*-$(h,r)$-concave operators, we establish the existence and uniqueness of solutions for system (1.1) dependent on two constants.

## Preliminaries

For the convenience of the reader, we present some concepts of Hadamard type fractional calculus to facilitate the analysis of system (1.1).

### Definition 2.1

(see [6])

For a function $g: [1,\infty)\rightarrow \mathbf{R}$, the Hadamard fractional integral of order *γ* is

$${}^{H} I^{\gamma}g(t)=\frac{1}{\Gamma(\gamma)} \int_{1}^{t} \biggl(\log\frac {t}{s} \biggr)^{\gamma-1}\frac{g(s)}{s}\,ds, \quad \gamma>0 $$

provided the integral exists.

### Definition 2.2

(see [6])

For a function $g:[1,\infty)\rightarrow \mathbf{R}$, the Hadamard fractional derivative of fractional order *γ* is

$${}^{H} D^{\gamma}g(t)=\frac{1}{\Gamma(n-\gamma)} \biggl(t \frac{d}{dt} \biggr)^{n} \int_{1}^{t} \biggl(\log\frac{t}{s} \biggr)^{n-\gamma-1}\frac{g(s)}{s}\,ds,\quad n-1< \gamma< n, n=[\gamma]+1, $$

where $[\gamma]$ denotes the integer part of the real number *γ* and $\log(\cdot)=\log_{e}(\cdot)$.

Set $\varrho_{q}(t)=(\log t)^{q-1}(1-\log t)$ and $\rho_{q}(t)=(1-\log t)^{q-1}\log t$ for $q>2$, $t\in[1,e]$, and

2.1 $$\begin{aligned} G_{q}(t,s)=\frac{1}{\Gamma(q)}\textstyle\begin{cases}(\log t)^{q-1}(1-\log s)^{q-1}-(\log(t/s))^{q-1},& 1\leq s\leq t\leq e,\\ (\log t)^{q-1}(1-\log s)^{q-1},& 1\leq t\leq s< e. \end{cases}\displaystyle \end{aligned}$$

### Lemma 2.1

(see [35])

*The function* $G_{q}(t,s)$ *in* (2.1) *has the following properties*:

(i) $G_{q}(t,s)$ *is continuous on* $(t,s)\in[1,e]^{2}$ *and* $G_{q}(t,s)>0$ *for* $t,s\in(1,e)$;
(ii) $\varrho_{q}(t)\rho_{q}(s)\leq\Gamma(q)G_{q}(t,s)\leq(q-1)\rho_{q}(s)$ *for* $t,s\in[1,e]$;
(iii) $\varrho_{q}(t)\rho_{q}(s)\leq\Gamma(q)G_{q}(t,s)\leq(q-1)\varrho_{q}(s)$ *for* $t,s\in[1,e]$.

*Next we also need some properties of the Green’s function to study system* (1.1).

### Lemma 2.2

(see [35])

*Let* $x,y\in C[0,1]$. *Then the following system*

2.2 $$\begin{aligned} \textstyle\begin{cases} {}^{H} D^{\alpha}u(t)+x(t)=0,\quad t\in(1,e),\\ {}^{H} D^{\beta}v(t)+y(t)=0,\quad t\in(1,e),\\ u^{(j)}(1)=v^{(j)}(1)=0,\quad 0\leq j\leq n-2,\\ u(e)=av(\xi),\qquad v(e)=bu(\eta),\quad \xi,\eta\in(1,e), \end{cases}\displaystyle \end{aligned}$$

*has an integral representation*

2.3 $$\begin{aligned} \textstyle\begin{cases} u(t)=\int_{1}^{e}K_{1}(t,s)\frac{x(s)}{s}\,ds+\int_{1}^{e}H_{1}(t,s)\frac {y(s)}{s}\,ds,\\ v(t)=\int_{1}^{e}K_{2}(t,s)\frac{y(s)}{s}\,ds+\int_{1}^{e}H_{2}(t,s)\frac{x(s)}{s}\,ds, \end{cases}\displaystyle \end{aligned}$$

*where*

2.4 $$\begin{aligned} &K_{1}(t,s)=G_{\alpha}(t,s)+\frac{ab(\log\xi)^{\beta-1}(\log t)^{\alpha -1}}{1-ab(\log\eta)^{\alpha-1}(\log\xi)^{\beta-1}}G_{\alpha}( \eta ,s), \end{aligned}$$

2.5 $$\begin{aligned} &K_{2}(t,s)=G_{\beta}(t,s)+\frac{ab(\log\eta)^{\alpha-1}(\log t)^{\beta -1}}{1-ab(\log\eta)^{\alpha-1}(\log\xi)^{\beta-1}}G_{\alpha}( \xi ,s), \end{aligned}$$

2.6 $$\begin{aligned} &H_{1}(t,s)=\frac{a(\log t)^{\alpha-1}}{1-ab(\log\eta)^{\alpha-1}(\log\xi )^{\beta-1}}G_{\beta}(\xi,s), \\ &H_{2}(t,s)=\frac{b(\log t)^{\beta-1}}{1-ab(\log\eta)^{\alpha-1}(\log \xi)^{\beta-1}}G_{\alpha}(\eta,s). \end{aligned}$$

### Lemma 2.3

(see [35])

*For* $t,s\in[1,e]$, *the functions* $K_{1}(t,s)$ *and* $H_{1}(t,s)$ *in* (2.4) *and* (2.6) *satisfy*

$$\begin{aligned} &\frac{ab(\log\xi)^{\beta-1}\varrho_{\alpha}(\eta)}{(1-ab(\log\eta )^{\alpha-1}(\log\xi)^{\beta-1})\Gamma(\alpha)}(\log t)^{\alpha-1}\rho _{\alpha}(s) \\ &\quad \leq K_{1}(t,s) \\ &\quad\leq\frac{ab(\log\xi)^{\beta-1}(1-(\log \eta)^{\alpha-1})}{(1-ab(\log\eta)^{\alpha-1}(\log \xi)^{\beta-1})\Gamma(\alpha-1)}\rho_{\alpha}(s), \\ &\frac{a\varrho_{\beta}(\xi)}{(1-ab(\log\eta)^{\alpha-1}(\log \xi)^{\beta-1})\Gamma(\beta)}(\log t)^{\alpha-1}\rho_{\beta}(s) \\ &\quad \leq H_{1}(t,s) \\ &\quad \leq\frac{a}{(1-ab(\log\eta)^{\alpha-1}(\log \xi)^{\beta-1})\Gamma(\beta-1)}\rho_{\beta}(s), \\ &K_{1}(t,s)\leq\frac{ab(\log\xi)^{\beta-1}(1-(\log \eta)^{\alpha-1})}{(1-ab(\log\eta)^{\alpha-1}(\log \xi)^{\beta-1})\Gamma(\alpha-1)}(\log t)^{\alpha-1}, \\ &H_{1}(t,s)\leq\frac{a}{(1-ab(\log\eta)^{\alpha-1}(\log \xi)^{\beta-1})\Gamma(\beta-1)}(\log t)^{\alpha-1}. \end{aligned}$$

### Lemma 2.4

(see [35])

*For* $t,s\in[1,e]$, *the functions* $K_{2}(t,s)$ *and* $H_{2}(t,s)$ *in* (2.5) *and* (2.6) *satisfy*

$$\begin{aligned} &\frac{ab(\log\eta)^{\beta-1}\varrho_{\beta}(\xi)}{(1-ab(\log\eta )^{\alpha-1}(\log\xi)^{\beta-1})\Gamma(\beta)}(\log t)^{\beta-1}\rho _{\beta}(s) \\ &\quad\leq K_{2}(t,s) \\ &\quad \leq\frac{ab(\log\eta)^{\alpha-1}(1-(\log \xi)^{\beta-1})}{(1-ab(\log\eta)^{\alpha-1}(\log \xi)^{\beta-1})\Gamma(\beta-1)}\rho_{\beta}(s), \\ &\frac{b\varrho_{\alpha}(\eta)}{(1-ab(\log\eta)^{\alpha-1}(\log \xi)^{\beta-1})\Gamma(\alpha)}(\log t)^{\beta-1}\rho_{\alpha}(s) \\ &\quad\leq H_{2}(t,s) \\ &\quad \leq\frac{b}{(1-ab(\log\eta)^{\alpha-1}(\log \xi)^{\beta-1})\Gamma(\alpha-1)}\rho_{\alpha}(s), \\ &K_{2}(t,s)\leq\frac{ab(\log\eta)^{\alpha-1}(1-(\log \xi)^{\beta-1})}{(1-ab(\log\eta)^{\alpha-1}(\log \xi)^{\beta-1})\Gamma(\alpha-1)}(\log t)^{\beta-1}, \\ &H_{2}(t,s)\leq\frac{b}{(1-ab(\log\eta)^{\alpha-1}(\log \xi)^{\beta-1})\Gamma(\alpha-1)}(\log t)^{\beta-1}. \end{aligned}$$

### Remark 2.1

(see [35])

For $t,s\in[1,e]$,

$$\begin{aligned} &\nu(\log t)^{\alpha-1}\rho_{\alpha}(s)\leq K_{1}(t,s)\leq \mu \rho_{\alpha}(s), \quad K_{1}(t,s)\leq\mu(\log t)^{\alpha-1}, \\ &\nu(\log t)^{\alpha-1}\rho_{\beta}(s)\leq H_{1}(t,s)\leq \mu \rho_{\beta}(s),\quad H_{1}(t,s)\leq\mu(\log t)^{\alpha-1}, \\ &\nu(\log t)^{\beta-1}\rho_{\beta}(s)\leq K_{2}(t,s)\leq\mu \rho_{\beta}(s),\quad K_{2}(t,s)\leq\mu(\log t)^{\beta-1}, \\ &\nu(\log t)^{\beta-1}\rho_{\alpha}(s)\leq H_{2}(t,s)\leq\mu \rho_{\alpha}(s), \quad H_{2}(t,s)\leq\mu(\log t)^{\beta-1}, \end{aligned}$$

where

$$\begin{aligned} \nu={}&\min \biggl\{ \frac{\min\{ab(\log \xi)^{\beta-1}\varrho_{\alpha}(\eta),b\varrho_{\alpha}(\eta)\}}{(1-ab(\log \eta)^{\alpha-1}(\log\xi)^{\beta-1})\Gamma(\alpha)}, \\ &\frac{\min\{ab(\log \eta)^{\alpha-1}\varrho_{\beta}(\xi),a\varrho_{\beta}(\xi)\}}{(1-ab(\log \eta)^{\alpha-1}(\log\xi)^{\beta-1})\Gamma(\beta)} \biggr\} , \\ \mu={}&\max \biggl\{ \frac{\max\{b,ab(\log\xi)^{\beta-1}(1-(\log \eta)^{\alpha-1})\}}{(1-ab(\log\eta)^{\alpha-1}(\log \xi)^{\beta-1})\Gamma(\alpha-1)}, \\ &\frac{\max\{a,ab(\log\eta)^{\alpha-1}(1-(\log \xi)^{\beta-1})\}}{(1-ab(\log\eta)^{\alpha-1}(\log \xi)^{\beta-1})\Gamma(\beta-1)} \biggr\} . \end{aligned}$$

Now we present a fixed point theorem which can be easily used to study some systems of differential equations.

Suppose that $(E,\|\cdot\|)$ is a real Banach space and it is partially ordered by a cone $P\subset E$. For any $x,y\in E$, $x\sim y$ denotes that there are $\psi>0$ and $\omega>0$ such that $\psi x\leq y\leq\omega x$. Take $h>\theta$ (i.e., $h\geq\theta$ and $h\neq\theta$), we consider a set $P_{h}=\{x\in E| x\sim h\}$. Clearly, $P_{h}\subset P$. Take another element $r\in P$ with $\theta\leq r\leq h$, we define $P_{h,r}=\{x\in E| x+r\in P_{h}\}$.

### Definition 2.3

(see [36])

Assume that $A:P_{h,r}\rightarrow E$ is an operator which satisfies: for any $x\in P_{h,r}$ and $\lambda\in (0,1)$, there exists $\varphi(\lambda)>\lambda$ such that $A(\lambda x+(\lambda-1)r)\geq\varphi(\lambda)Ax+(\varphi(\lambda)-1)r$. Then we call *A* a *φ*-$(h,r)$-concave operator.

### Lemma 2.5

(see [36])

*Suppose that* *P* *is normal and* *A* *is an increasing* *φ*-$(h,r)$-*concave operator satisfying* $Ah\in P_{h,r}$. *Then* *A* *has a unique fixed point* $x^{*}$ *in* $P_{h,r}$. *In addition*, *for any* $w_{0}\in P_{h,r}$, *construct the sequence* $w_{n}=Aw_{n-1}$, $n=1,2,\ldots$ , *then* $\|w_{n}-x^{*}\|\rightarrow0$ *as* $n\rightarrow\infty$.

For $h_{1},h_{2}\in P$ with $h_{1},h_{2}\neq\theta$. Let $h=(h_{1},h_{2})$, then $h\in\overline{P}:=P\times P$. Take $\theta\leq r_{1}\leq h_{1}$, $\theta \leq r_{2}\leq h_{2}$, and let $\overline{\theta}=(\theta,\theta), r=(r_{1},r_{2})$. Then $\overline{\theta}=(\theta,\theta)\leq(r_{1},r_{2})\leq (h_{1},h_{2})=h$. That is, $\overline{\theta}\leq r\leq h$. If *P* is normal, then $\overline{P}=P\times P$ is normal (see [37]).

### Lemma 2.6

(see [38])

$\overline{P_{h}}=P_{h_{1}}\times P_{h_{2}}$.

### Lemma 2.7

(see [39])

$\overline{P}_{h,r}=P_{{h_{1},r_{1}}}\times P_{{h_{2}},r_{2}}$.

## Existence and uniqueness of solutions

In this section, let $E=C[1,e]$, then *E* is a Banach space with the norm $\|u\|=\max_{t\in[1,e]} |u(t)|$. We will consider (1.1) in $E\times E$. For $(u,v)\in E\times E$, let $\|(u,v)\|=\max\{\|u\|,\|v\| \}$. It is clear that $(E\times E,\|(\cdot,\cdot)\|)$ is a Banach space. Let $\overline{P}=\{(u,v)\in E\times E|u(t)\geq0,v(t)\geq0\}$, $P=\{u\in E\mid u(t)\geq0,t\in[1,e]\}$, then the cone $\overline {P}\subset E\times E$ and $\overline{P}=P\times P$ is normal, and the space $E\times E$ has a partial order: $(u_{1},v_{1})\leq (u_{2},v_{2})\Leftrightarrow u_{1}(t)\leq u_{2}(t),v_{1}(t)\leq v_{2}(t),t\in [1,e]$.

Suppose $f(t,x),g(t,x)$ are continuous, from Lemma 2.2, $(u,v)\in E\times E$ is a solution of (1.1) if and only if $(u,v)\in E\times E$ is a solution of the following equations:

$$\textstyle\begin{cases} u(t)=\int_{1}^{e}K_{1}(t,s)f(s,v(s))\frac{ds}{s}+\int _{1}^{e}H_{1}(t,s)g(s,u(s))\frac{ds}{s}-l_{f}\int_{1}^{e}(K_{1}(t,s)+H_{1}(t,s))\frac {ds}{s},\\ v(t)=\int_{1}^{e}K_{2}(t,s)g(s,u(s))\frac{ds}{s}+\int _{1}^{e}H_{2}(t,s)f(s,v(s))\frac{ds}{s}-l_{g}\int_{1}^{e}(K_{2}(t,s)+H_{2}(t,s))\frac{ds}{s}. \end{cases} $$

For $(u,v)\in E\times E$, we define three operators $A_{1}$, $A_{2}$, and *T* by

$$\begin{aligned} &A_{1}u(t)= \int_{1}^{e}K_{1}(t,s)f \bigl(s,v(s) \bigr) \frac{ds}{s}+ \int _{1}^{e}H_{1}(t,s)g \bigl(s,u(s) \bigr)\frac{ds}{s}-l_{f} \int_{1}^{e} \bigl(K_{1}(t,s)+H_{1}(t,s) \bigr)\frac{ds}{s}, \\ &A_{2}v(t)= \int_{1}^{e}K_{2}(t,s)g \bigl(s,u(s) \bigr) \frac{ds}{s}+ \int _{1}^{e}H_{2}(t,s)f \bigl(s,v(s) \bigr) \frac{ds}{s}-l_{g} \int_{1}^{e} \bigl(K_{2}(t,s)+H_{2}(t,s) \bigr)\frac{ds}{s}, \end{aligned}$$

and $T(u,v)(t)=(A_{1}u(t),A_{2}v(t))$. Then $A_{1},A_{2}:E\rightarrow E$ and $T:E\times E\rightarrow E\times E$. Evidently, $(u,v)$ is the solution of system (1.1) if and only if $(u,v)$ is the fixed point of operator *T*. Let

3.1 $$\begin{aligned} &r_{1}(t)=l_{f} \int_{1}^{e} \bigl(K_{1}(t,s)+H_{1}(t,s) \bigr)\frac{ds}{s}, \qquad r_{2}(t)=l_{g} \int _{1}^{e} \bigl(K_{2}(t,s)+H_{2}(t,s) \bigr)\frac{ds}{s}, \end{aligned}$$

3.2 $$\begin{aligned} & h_{1}(t)=M_{1}(\log t)^{\alpha-1},\qquad h_{2}(t)=M_{2}( \log t)^{\beta-1},\quad t\in [1,e], \end{aligned}$$

where $M_{1}\geq2\mu l_{f}$, $M_{2}\geq2\mu l_{g}$.

### Theorem 3.1

*Let* $\alpha,\beta\in(n-1,n]$, $l_{f}>0, l_{g}>0$, *and* $r_{1},r_{2},h_{1},h_{2}$ *be given as in* (3.1), (3.2). *Assume that* $f,g\in C([1,e]\times(-\infty,+\infty),(-\infty,+\infty))$; *moreover*,

$(H_{1})$
$f:[1,e]\times[-r_{2}^{*},+\infty)\rightarrow(-\infty,+\infty)$ *is increasing with respect to the second variable*, *where* $r_{2}^{*}=\max\{ r_{2}(t):t\in[1,e]\}$; $g:[1,e]\times[-r_{1}^{*},+\infty)\rightarrow(-\infty ,+\infty)$ *is increasing with respect to the second variable*, *where* $r_{1}^{*}=\max\{r_{1}(t):t\in[1,e]\}$;
$(H_{2})$
*for* $\lambda\in(0,1)$, *there exists* $\varphi(\lambda)>\lambda$ *such that*
  $$\begin{aligned} &f \bigl(t,\lambda x+(\lambda-1)y \bigr)\geq\varphi(\lambda)f(t,x),\quad t \in[1,e],x\in (-\infty,+\infty),y\in \bigl[0,r_{2}^{*} \bigr], \\ &g \bigl(t,\lambda x+(\lambda-1)y \bigr)\geq\varphi(\lambda)g(t,x),\quad t \in[1,e],x\in (-\infty,+\infty),y\in \bigl[0,r_{1}^{*} \bigr]; \end{aligned}$$
$(H_{3})$
$f(t,0)\geq0, g(t,0)\geq0$ *with* $f(t,0)\not\equiv0,g(t,0)\not \equiv0$ *for* $t\in[1,e]$.

*Then*:

1. *system* (1.1) *has a unique solution* $(u^{*},v^{*})$ *in* $\overline {P}_{h,r}$, *where*
  $$r(t)= \bigl(r_{1}(t),r_{2}(t) \bigr),\qquad h(t)= \bigl(h_{1}(t),h_{2}(t) \bigr), \quad t\in[1,e]; $$
2. *for a given point* $(u_{0},v_{0})\in\overline{P}_{h,r}$, *construct the following sequences*:
  $$\begin{aligned} &u_{n+1}(t)= \int_{1}^{e}K_{1}(t,s)f \bigl(s,v_{n}(s) \bigr)\frac{ds}{s}+ \int _{1}^{e}H_{1}(t,s)g \bigl(s,u_{n}(s) \bigr)\frac{ds}{s}\\ &\phantom{u_{n+1}(t)=}{}-l_{f} \int_{1}^{e} \bigl(K_{1}(t,s)+H_{1}(t,s) \bigr)\frac{ds}{s}, \\ &v_{n+1}(t)= \int_{1}^{e}K_{2}(t,s)g \bigl(s,u_{n}(s) \bigr)\frac{ds}{s}+ \int _{1}^{e}H_{2}(t,s)f \bigl(s,v_{n}(s) \bigr)\frac{ds}{s}\\ &\phantom{v_{n+1}(t)=}{}-l_{g} \int_{1}^{e} \bigl(K_{2}(t,s)+H_{2}(t,s) \bigr)\frac{ds}{s}, \end{aligned}$$
  $n=0,1,2,\ldots$ , *we have* $u_{n+1}(t)\rightarrow u^{*}(t)$, $v_{n+1}(t)\rightarrow v^{*}(t)$ *as* $n\rightarrow\infty$.

### Proof

By Lemma 2.1, for $t\in[1,e]$,

$$r_{1}(t)=l_{f} \int_{1}^{e} \bigl(K_{1}(t,s)+H_{1}(t,s) \bigr)\frac{ds}{s}\geq0,\qquad r_{2}(t)=l_{g} \int _{1}^{e} \bigl(K_{2}(t,s)+H_{2}(t,s) \bigr)\frac{ds}{s}\geq0. $$

From Remark 2.1, for $t\in[1,e]$,

$$\begin{aligned} r_{1}(t)&=l_{f} \int_{1}^{e} \bigl(K_{1}(t,s)+H_{1}(t,s) \bigr)\frac{ds}{s} \\ &\leq l_{f} \int_{1}^{e}2\mu(\log t)^{\alpha-1} \frac{ds}{s} \\ &= 2\mu l_{f}(\log t)^{\alpha-1} \int_{1}^{e}\frac{ds}{s} \\ &\leq M_{1}(\log t)^{\alpha-1}=h_{1}(t), \\ r_{2}(t)&=l_{g} \int_{1}^{e} \bigl(K_{2}(t,s)+H_{2}(t,s) \bigr)\frac{ds}{s} \\ &\leq l_{g} \int_{1}^{e}2\mu(\log t)^{\beta-1} \frac{ds}{s} \\ &= 2\mu l_{g}(\log t)^{\beta-1} \int_{1}^{e}\frac{ds}{s} \\ &\leq M_{2}(\log t)^{\beta-1}=h_{2}(t). \end{aligned}$$

That is, $0\leq r_{1}\leq h_{1}$, $0\leq r_{2}\leq h_{2}$.

In the following, we prove that $T:\overline{P}_{h,r}\rightarrow E\times E$ is a *φ*-$(h,r)$-concave operator. For $(u,v)\in \overline{P}_{h,r}$, $\lambda\in(0,1)$, we obtain

$$\begin{aligned} T \bigl(\lambda(u,v)+(\lambda-1)r \bigr) (t) =&T \bigl(\lambda(u,v)+(\lambda-1) (r_{1},r_{2}) \bigr) (t) \\ =&T \bigl(\lambda u+(\lambda-1)r_{1},\lambda v+( \lambda-1)r_{2} \bigr) (t) \\ =& \bigl(A_{1} \bigl(\lambda u+(\lambda-1)r_{1} \bigr) \bigr),A_{2} \bigl(\lambda v+(\lambda-1)r_{2} \bigr)) (t). \end{aligned}$$

We discuss $A_{1}(\lambda u+(\lambda-1)r_{1})(t)$ and $A_{2}(\lambda v+(\lambda-1)r_{2})(t)$, respectively. From $(H_{2})$,

$$\begin{aligned} & A_{1} \bigl(\lambda u+(\lambda-1)r_{1} \bigr) (t) \\ &\quad = \int_{1}^{e}K_{1}(t,s)f \bigl(s,\lambda v(s)+(\lambda-1)r_{2}(s) \bigr)\frac{ds}{s}\\ &\qquad{}+ \int _{1}^{e}H_{1}(t,s)g \bigl(s,\lambda u(s)+(\lambda-1)r_{1}(s) \bigr)\frac{ds}{s}-r_{1}(t) \\ &\quad \geq \varphi(\lambda) \biggl[ \int_{1}^{e}K_{1}(t,s)f \bigl(s,v(s) \bigr) \frac{ds}{s}+ \int _{1}^{e}H_{1}(t,s)g \bigl(s,u(s) \bigr)\frac{ds}{s} \biggr]-r_{1}(t) \\ &\quad = \varphi(\lambda) \biggl[ \int_{1}^{e}K_{1}(t,s)f \bigl(s,v(s) \bigr) \frac{ds}{s}+ \int _{1}^{e}H_{1}(t,s)g \bigl(s,u(s) \bigr)\frac{ds}{s}-r_{1}(t) \biggr]+ \bigl[\varphi(\lambda )-1 \bigr]r_{1}(t) \\ &\quad = \varphi(\lambda)A_{1}u(t)+ \bigl[\varphi(\lambda)-1 \bigr]r_{1}(t), \\ &A_{2} \bigl(\lambda v+(\lambda-1)r_{2} \bigr) (t) \\ &\quad = \int_{1}^{e}K_{2}(t,s)g \bigl(s,\lambda u(s)+(\lambda-1)r_{1}(s) \bigr)\frac{ds}{s}\\ &\qquad{}+ \int _{1}^{e}H_{2}(t,s)f \bigl(s,\lambda v(s)+(\lambda-1)r_{2}(s) \bigr)\frac{ds}{s}-r_{2}(t) \\ &\quad \geq \varphi(\lambda) \biggl[ \int_{1}^{e}K_{2}(t,s)g \bigl(s,u(s) \bigr) \frac{ds}{s}+ \int _{1}^{e}H_{2}(t,s)f \bigl(s,v(s) \bigr) \frac{ds}{s} \biggr]-r_{2}(t) \\ &\quad = \varphi(\lambda) \biggl[ \int_{1}^{e}K_{2}(t,s)g \bigl(s,u(s) \bigr) \frac{ds}{s}+ \int _{1}^{e}H_{2}(t,s)f \bigl(s,v(s) \bigr) \frac{ds}{s}-r_{2}(t) \biggr]\\ &\qquad{}+ \bigl[\varphi(\lambda )-1 \bigr]r_{2}(t) \\ &\quad = \varphi(\lambda)A_{2}v(t)+ \bigl[\varphi(\lambda)-1 \bigr]r_{2}(t). \end{aligned}$$

So we have

$$\begin{aligned} & T \bigl(\lambda(u,v)+(\lambda-1)r \bigr) (t) \\ &\quad \geq \bigl(\varphi(\lambda)A_{1}u(t)+ \bigl[\varphi(\lambda)-1 \bigr]r_{1}(t),\varphi (\lambda)A_{2}v(t)+ \bigl[\varphi( \lambda)-1 \bigr]r_{2}(t) \bigr) \\ &\quad = \bigl(\varphi(\lambda)A_{1}u(t),\varphi( \lambda)A_{2}v(t) \bigr)+ \bigl( \bigl(\varphi(\lambda )-1 \bigr)r_{1}(t), \bigl(\varphi( \lambda)-1 \bigr)r_{2}(t) \bigr) \\ &\quad = \varphi(\lambda) \bigl(A_{1}u(t),A_{2}v(t) \bigr)+ \bigl( \varphi(\lambda )-1 \bigr) \bigl(r_{1}(t),r_{2}(t) \bigr) \\ &\quad = \varphi(\lambda)T(u,v) (t)+ \bigl(\varphi(\lambda)-1 \bigr))r(t). \end{aligned}$$

That is,

$$T \bigl(\lambda(u,v)+(\lambda-1)r \bigr)\geq\varphi(\lambda)T(u,v)+ \bigl[ \varphi( \lambda )-1) \bigr]r,\quad (u,v)\in\overline{P}_{h,r},\lambda \in(0,1). $$

Hence, *T* is a *φ*-$(h,r)$-concave operator.

Next we show that $T:\overline{P}_{h,r}\rightarrow E\times E$ is increasing. For $(u,v)\in\overline{P}_{h,r}$, we have $(u,v)+r\in \overline{P}_{h}$. From Lemma 2.6, $(u+r_{1},v+r_{2})\in P_{h_{1}}\times P_{h_{2}}$. So there are $\lambda_{1},\lambda_{2}>0$ such that

$$u(t)+r_{1}(t)\geq\lambda_{1}h_{1}(t),\qquad v(t)+r_{2}(t)\geq\lambda_{2}h_{2}(t),\quad t \in[1,e]. $$

Therefore, $u(t)\geq\lambda_{1}h_{1}(t)-r_{1}(t)\geq-r_{1}(t)\geq-r_{1}^{*}, v(t)\geq\lambda_{2}h_{2}(t)-r_{2}(t)\geq-r_{2}(t)\geq-r_{2}^{*}$. By $(H_{1})$ and the definitions of $A_{1},A_{2}$, we obtain $T:\overline {P}_{h,r}\rightarrow E\times E$ is increasing.

Now we prove that $Th\in\overline{P}_{h,r}$, so we need to prove $Th+r\in\overline{P}_{h}$. For $t\in[1,e]$,

$$\begin{aligned} & Th(t)+r(t) \\ &\quad =T(h_{1},h_{2}) (t)+r(t)= \bigl(A_{1}h_{1}(t),A_{2}h_{2}(t) \bigr)+ \bigl(r_{1}(t),r_{2}(t) \bigr) \\ &\quad = \bigl(A_{1}h_{1}(t)+r_{1}(t),A_{2}h_{2}(t)+r_{2}(t) \bigr). \end{aligned}$$

We discuss $A_{1}h_{1}(t)+r_{1}(t),A_{2}h_{2}(t)+r_{2}(t)$, respectively. By Remark 2.1 and $(H_{1})$, $(H_{3})$,

$$\begin{aligned} & A_{1}h_{1}(t)+r_{1}(t) \\ &\quad = \int_{1}^{e}K_{1}(t,s)f \bigl(s,h_{2}(s) \bigr)\frac{ds}{s}+ \int _{1}^{e}H_{1}(t,s)g \bigl(s,h_{1}(s) \bigr)\frac{ds}{s} \\ &\quad \geq \int_{1}^{e}\nu(\log t)^{\alpha-1} \rho_{\alpha}(s)f \bigl(s,M_{2}(\log s)^{\beta -1} \bigr) \frac{ds}{s}\\ &\qquad{}+ \int_{1}^{e}\nu(\log t)^{\alpha-1} \rho_{\beta}(s)g \bigl(s,M_{1}(\log s)^{\alpha-1} \bigr) \frac{ds}{s} \\ &\quad \geq \nu(\log t)^{\alpha-1} \int_{1}^{e}\rho_{\alpha}(s)f(s,0) \frac{ds}{s}+\nu (\log t)^{\alpha-1} \int_{1}^{e}\rho_{\beta}(s)g(s,0) \frac{ds}{s} \\ &\quad = \frac{\nu}{M_{1}} \int_{1}^{e} \bigl(\rho_{\alpha}(s)f(s,0)+ \rho_{\beta}(s)g(s,0) \bigr)\frac{ds}{s}\cdot h_{1}(t), \\ &A_{1}h_{1}(t)+r_{1}(t) \\ &\quad = \int_{1}^{e}K_{1}(t,s)f \bigl(s,h_{2}(s) \bigr)\frac{ds}{s}+ \int _{1}^{e}H_{1}(t,s)g \bigl(s,h_{1}(s) \bigr)\frac{ds}{s} \\ &\quad \leq \int_{1}^{e}\mu(\log t)^{\alpha-1}f(s,M_{2}) \frac{ds}{s}+ \int_{1}^{e}\mu (\log t)^{\alpha-1}g(s,M_{1}) \frac{ds}{s} \\ &\quad = \mu(\log t)^{\alpha-1} \int_{1}^{e} \bigl(f(s,M_{2})+g(s,M_{1}) \bigr)\frac{ds}{s} \\ &\quad = \frac{\mu}{M_{1}} \int_{1}^{e} \bigl(f(s,M_{2})+g(s,M_{1}) \bigr)\frac{ds}{s}\cdot h_{1}(t). \end{aligned}$$

From $(H_{1})$, $(H_{3})$, one has $\int_{1}^{e}(f(s,M_{2})+g(s,M_{1}))\frac {ds}{s}\geq\int_{1}^{e}(\rho_{\alpha}(s)f(s,0)+\rho_{\beta}(s)g(s,0))\frac {ds}{s}>0$. Let

$$\begin{aligned} &l_{1}:=\frac{\nu}{M_{1}} \int_{1}^{e} \bigl(\rho_{\alpha}(s)f(s,0)+ \rho_{\beta}(s)g(s,0) \bigr)\frac{ds}{s}, \\ &l_{2}:=\frac{\mu}{M_{1}} \int_{1}^{e} \bigl(f(s,M_{2})+g(s,M_{1}) \bigr)\frac{ds}{s}. \end{aligned}$$

Note that $\rho_{\alpha}(s)\leq1$, $\rho_{\beta}(s)\leq1$, so $l_{1}\leq l_{2}$, and thus $l_{1}h_{1}(t)\leq A_{1}h_{1}(t)+r_{1}(t)\leq l_{2}h_{1}(t)$. This shows $A_{1}h_{1}+r_{1}\in P_{h_{1}}$. Similarly, we can also get $A_{2}h_{2}+r_{2}\in P_{h_{2}}$. Consequently, by Lemma 2.7,

$$Th+r=(A_{1}h_{1}+r_{1},A_{2}h_{2}+r_{2}) \in P_{h_{1}}\times P_{h_{2}}=\overline{P}_{h}. $$

Finally, by using Lemma 2.5, *T* has a unique fixed point $(u^{*},v^{*})\in \overline{P}_{h,r}$. In addition, for any given $(u_{0},v_{0})\in\overline {P}_{h,r}$, the sequence

$$(u_{n},v_{n})=(A_{1}u_{n-1},A_{2}v_{n-1}),\quad n=1,2,\ldots $$

converges to $(u^{*},v^{*})$ as $n\rightarrow\infty$. Therefore, system (1.1) has a unique solution $(u^{*},v^{*})$ in $\overline{P}_{h,r}$; taking any point $(u_{0},v_{0})\in\overline{P}_{h,r}$, construct the following sequences:

$$\begin{aligned} u_{n+1}(t)={}& \int_{1}^{e}K_{1}(t,s)f \bigl(s,v_{n}(s) \bigr)\frac{ds}{s}+ \int _{1}^{e}H_{1}(t,s)g \bigl(s,u_{n}(s) \bigr)\frac{ds}{s}\\ &{}-l_{f} \int_{1}^{e} \bigl(K_{1}(t,s)+H_{1}(t,s) \bigr)\frac{ds}{s}, \\ v_{n+1}(t)={}& \int_{1}^{e}K_{2}(t,s)g \bigl(s,u_{n}(s) \bigr)\frac{ds}{s}+ \int _{1}^{e}H_{2}(t,s)f \bigl(s,v_{n}(s) \bigr)\frac{ds}{s}\\ &{}-l_{g} \int_{1}^{e} \bigl(K_{2}(t,s)+H_{2}(t,s) \bigr)\frac{ds}{s}, \end{aligned}$$

$n=0,1,2,\ldots$ , we have $u_{n+1}(t)\rightarrow u^{*}(t),v_{n+1}(t)\rightarrow v^{*}(t)$ as $n\rightarrow\infty$. □

## Example

We consider the following Hadamard fractional boundary value problem:

4.1 $$\begin{aligned} \textstyle\begin{cases} {}^{H} D^{\frac{5}{2}} u(t)+ (\frac{25\sqrt {15}\cdot k^{\frac{3}{2}}}{18\cdot k_{2}^{\frac{5}{2}}}v(t)+1 )^{\frac{1}{5}} (k_{2}-k\log t )^{\frac{1}{5}}(\log t)^{\frac {3}{10}}=1,\quad t\in(1,e),\\ {}^{H} D^{\frac{5}{2}} v(t)+ (\frac{25\sqrt{15}\cdot k^{\frac {3}{2}}}{18\cdot k_{1}^{\frac{5}{2}}}u(t)+1 )^{\frac{1}{5}} (k_{1}-k\log t )^{\frac{1}{5}}(\log t)^{\frac{3}{10}}=1,\quad t\in(1,e),\\ u(1)=v(1)=u'(1)=v'(1)=0,\\ u(e)=v(e^{\frac{1}{2}}),\qquad v(e)=2u(e^{\frac{1}{3}}), \end{cases}\displaystyle \end{aligned}$$

where $\alpha=\beta=\frac{5}{2}$, $n=3$, $a=1$, $b=2$, $\xi=e^{\frac {1}{2}}$, $\eta=e^{\frac{1}{3}}$ with $0< ab(\log\eta)^{\alpha-1}(\log \xi)^{\beta-1}=1\times2\times(\log e^{\frac{1}{3}})^{\frac{3}{2}}\times (\log e^{\frac{1}{2}})^{\frac{3}{2}}=\frac{\sqrt{6}}{18}<1$, and

$$\begin{aligned} &k_{1}=\frac{8\sqrt{\pi}}{15\pi}+\frac{432\sqrt{6\pi}+144\sqrt{\pi }}{21{,}465}+\frac{54\sqrt{2\pi}+6\sqrt{3\pi}}{795\pi}, \\ &k_{2}=\frac{8\sqrt{\pi}}{15\pi}+\frac{288\sqrt{2\pi}+1728\sqrt{3\pi }}{21{,}465}+\frac{12\sqrt{6\pi}+4\sqrt{\pi}}{795\pi},\quad k= \frac{8\sqrt{\pi }}{15\pi}, \\ &f(t,x)= \biggl(\frac{25\sqrt{15}\cdot k^{\frac{3}{2}}}{18\cdot k_{2}^{\frac {5}{2}}}x+1 \biggr)^{\frac{1}{5}} (k_{2}-k \log t )^{\frac {1}{5}}(\log t)^{\frac{3}{10}}, \\ &g(t,x)= \biggl(\frac{25\sqrt{15}\cdot k^{\frac{3}{2}}}{18\cdot k_{1}^{\frac {5}{2}}}x+1 \biggr)^{\frac{1}{5}} (k_{1}-k \log t )^{\frac {1}{5}}(\log t)^{\frac{3}{10}}, \end{aligned}$$

$l_{f}=l_{g}=1$. Obviously, $k_{1}, k_{2}>k$, and

$$\begin{aligned} &f(t,0)=(k_{2}-k\log t)^{\frac{1}{5}}(\log t)^{\frac{3}{10}}\geq0, \\ &g(t,0)=(k_{1}-k\log t)^{\frac{1}{5}}(\log t)^{\frac{3}{10}}\geq0, \end{aligned}$$

with $f(t,0)\not\equiv0, g(t,0)\not\equiv0$. And $\varrho_{\alpha}(\eta)=(\frac{1}{3})^{\frac{3}{2}}\times(1-\frac {1}{3})=\frac{2\sqrt{3}}{27}$, $\varrho_{\beta}(\xi)=(\frac{1}{2})^{\frac {3}{2}}(1-\frac{1}{2})=\frac{\sqrt{2}}{8}$.

$$\begin{aligned} &G_{\alpha}(t,s)=\frac{1}{\Gamma(\frac{5}{2})} \textstyle\begin{cases} (\log t)^{\frac{3}{2}}(1-\log s)^{\frac{3}{2}}-(\log(t/s))^{\frac{3}{2}},& 1\leq s\leq t\leq e,\\ (\log t)^{\frac{3}{2}}(1-\log s)^{\frac{3}{2}},& 1\leq t\leq s< e, \end{cases}\displaystyle \\ &G_{\alpha}(\eta,s)=\frac{1}{\Gamma(\frac{5}{2})} \textstyle\begin{cases} (\frac{1}{3})^{\frac{3}{2}}(1-\log s)^{\frac {3}{2}}-(\frac{1}{3}-\log s)^{\frac{3}{2}},& 1\leq s\leq\eta\leq e,\\ (\frac{1}{3})^{\frac{3}{2}}(1-\log s)^{\frac{3}{2}},& 1\leq\eta\leq s< e, \end{cases}\displaystyle \\ &G_{\beta}(\xi,s)=\frac{1}{\Gamma(\frac{5}{2})} \textstyle\begin{cases} (\frac{1}{2})^{\frac{3}{2}}(1-\log s)^{\frac {3}{2}}-(\frac{1}{2}-\log s)^{\frac{3}{2}},& 1\leq s\leq\xi\leq e,\\ (\frac{1}{2})^{\frac{3}{2}}(1-\log s)^{\frac{3}{2}},& 1\leq\xi\leq s< e, \end{cases}\displaystyle \\ &G_{\beta}(t,s)=\frac{1}{\Gamma(\frac{5}{2})} \textstyle\begin{cases} (\log t)^{\frac{3}{2}}(1-\log s)^{\frac{3}{2}}-(\log(t/s))^{\frac{3}{2}},& 1\leq s\leq t\leq e,\\ (\log t)^{\frac{3}{2}}(1-\log s)^{\frac{3}{2}},& 1\leq t\leq s< e, \end{cases}\displaystyle \\ &G_{\alpha}(\xi,s)=\frac{1}{\Gamma(\frac{5}{2})} \textstyle\begin{cases} (\frac{1}{2})^{\frac{3}{2}}(1-\log s)^{\frac {3}{2}}-(\frac{1}{2}-\log s)^{\frac{3}{2}},& 1\leq s\leq\xi\leq e,\\ (\frac{1}{2})^{\frac{3}{2}}(1-\log s)^{\frac{3}{2}},& 1\leq\xi\leq s< e, \end{cases}\displaystyle \\ &\nu = \min \biggl\{ \frac{\min\{ab(\log \xi)^{\beta-1}\varrho_{\alpha}(\eta),b\varrho_{\alpha}(\eta)\}}{(1-ab(\log \eta)^{\alpha-1}(\log\xi)^{\beta-1})\Gamma(\alpha)},\frac{\min\{ab(\log \eta)^{\alpha-1}\varrho_{\beta}(\xi),a\varrho_{\beta}(\xi)\}}{(1-ab(\log \eta)^{\alpha-1}(\log\xi)^{\beta-1})\Gamma(\beta)} \biggr\} \\ &\phantom{\nu}= \min \biggl\{ \frac{\min\{1\times2\times(\frac{1}{2})^{\frac {3}{2}}\times\frac{2\sqrt{3}}{27},2\times\frac{2\sqrt{3}}{27}\} }{[1-1\times2\times(\frac{1}{3})^{\frac{3}{2}}\times(\frac{1}{2})^{\frac {3}{2}}]\Gamma(\frac{5}{2})} ,\frac{\min\{1\times2\times(\frac{1}{3})^{\frac{3}{2}}\times\frac{\sqrt {2}}{8},1\times\frac{\sqrt{2}}{8}\}}{[1-1\times2\times(\frac {1}{3})^{\frac{3}{2}}\times(\frac{1}{2})^{\frac{3}{2}}]\Gamma(\frac {5}{2})} \biggr\} \\ &\phantom{\nu}= \min \biggl\{ \frac{\min\{\frac{\sqrt{6}}{27},\frac{4\sqrt{3}}{27}\} }{\frac{54\sqrt{\pi}-3\sqrt{6\pi}}{72}},\frac{\min\{\frac{\sqrt {6}}{36},\frac{\sqrt{2}}{8}\}}{\frac{54\sqrt{\pi}-3\sqrt{6\pi}}{72}} \biggr\} \\ &\phantom{\nu}= \frac{\sqrt{\pi}(6\sqrt{6}+2)}{159\pi}, \\ &\mu = \max \biggl\{ \frac{\max\{b,ab(\log\xi)^{\beta-1}(1-(\log \eta)^{\alpha-1})\}}{(1-ab(\log\eta)^{\alpha-1}(\log \xi)^{\beta-1})\Gamma(\alpha-1)},\frac{\max\{a,ab(\log\eta)^{\alpha -1}(1-(\log \xi)^{\beta-1})\}}{(1-ab(\log\eta)^{\alpha-1}(\log \xi)^{\beta-1})\Gamma(\beta-1)} \biggr\} \\ &\phantom{\mu}= \max \biggl\{ \frac{\max\{2,1\times2\times(\frac{1}{2})^{\frac {3}{2}}\times(1-(\frac{1}{3})^{\frac{3}{2}})\}}{[1-1\times2\times(\frac {1}{3})^{\frac{3}{2}}\times(\frac{1}{2})^{\frac{3}{2}}]\Gamma(\frac{5}{2}-1)} ,\frac{\max\{1,1\times2\times(\frac{1}{3})^{\frac{3}{2}}\times(1-(\frac {1}{2})^{\frac{3}{2}})\}}{[1-1\times2\times(\frac{1}{3})^{\frac {3}{2}}\times(\frac{1}{2})^{\frac{3}{2}}]\Gamma(\frac{5}{2}-1)} \biggr\} \\ &\phantom{\mu}= \max \biggl\{ \frac{\max\{2,\frac{9\sqrt{2}-\sqrt{6}}{18}\}}{\frac {18\sqrt{\pi}-\sqrt{6\pi}}{36}},\frac{\max\{1,\frac{4\sqrt{3}-\sqrt {6}}{18}\}}{\frac{18\sqrt{\pi}-\sqrt{6\pi}}{36}} \biggr\} \\ &\phantom{\mu}= \frac{12\sqrt{\pi}(\sqrt{6}+18)}{53\pi}. \end{aligned}$$

Further,

$$\begin{aligned} r_{1}(t)={}&l_{f} \int_{1}^{e} \bigl(K_{1}(t,s)+H_{1}(t,s) \bigr)\frac{ds}{s} \\ ={}& \int_{1}^{e} G_{\alpha}(t,s) \frac{ds}{s}+\frac{ab(\log\xi)^{\beta -1}(\log t)^{\alpha-1}}{1-ab(\log\eta)^{\alpha-1}(\log\xi)^{\beta -1}} \int_{1}^{e}G_{\alpha}(\eta,s) \frac{ds}{s} \\ & {} +\frac{a(\log t)^{\alpha-1}}{1-ab(\log\eta)^{\alpha-1}(\log\xi )^{\beta-1}} \int_{1}^{e}G_{\beta}(\xi,s) \frac{ds}{s} \\ ={}& \int_{1}^{e} G_{\alpha}(t,s) \frac{ds}{s}+\frac{27\sqrt{2}+3\sqrt {3}}{53}\cdot(\log t)^{\frac{3}{2}} \int_{1}^{e}G_{\alpha}(\eta,s) \frac {ds}{s}\\ &{}+\frac{54+3\sqrt{6}}{53}\cdot(\log t)^{\frac{3}{2}} \int _{1}^{e}G_{\beta}(\xi,s) \frac{ds}{s} \\ ={}& \biggl(\frac{8\sqrt{\pi}}{15\pi}+\frac{432\sqrt{6\pi}+144\sqrt{\pi }}{21{,}465}+\frac{54\sqrt{2\pi}+6\sqrt{3\pi}}{795\pi} \biggr) \cdot(\log t)^{\frac{3}{2}}-\frac{8\sqrt{\pi}}{15\pi}\cdot(\log t)^{\frac{5}{2}}, \\ r_{2}(t)={}&l_{g} \int_{1}^{e} \bigl(K_{2}(t,s)+H_{2}(t,s) \bigr)\frac{ds}{s} \\ ={}& \int_{1}^{e}G_{\beta}(t,s)\frac{ds}{s}+ \frac{ab(\log\eta)^{\alpha -1}(\log t)^{\beta-1}}{1-ab(\log\eta)^{\alpha-1}(\log\xi)^{\beta -1}} \int_{1}^{e}G_{\alpha}(\xi,s) \frac{ds}{s} \\ & {} +\frac{b(\log t)^{\beta-1}}{1-ab(\log\eta)^{\alpha-1}(\log \xi)^{\beta-1}} \int_{1}^{e}G_{\alpha}(\eta,s) \frac{ds}{s} \\ ={}& \int_{1}^{e}G_{\beta}(t,s)\frac{ds}{s}+ \frac{12\sqrt{3}+2\sqrt {2}}{53}\cdot(\log t)^{\frac{3}{2}} \int_{1}^{e}G_{\alpha}(\xi,s) \frac {ds}{s}\\ &{}+\frac{108+6\sqrt{6}}{53}\cdot(\log t)^{\frac{3}{2}} \int _{1}^{e}G_{\alpha}(\eta,s) \frac{ds}{s} \\ ={}& \biggl(\frac{8\sqrt{\pi}}{15\pi}+\frac{288\sqrt{2\pi}+1728\sqrt{3\pi }}{21{,}465}+\frac{12\sqrt{6\pi}+4\sqrt{\pi}}{795\pi} \biggr) \cdot(\log t)^{\frac{3}{2}}-\frac{8\sqrt{\pi}}{15\pi}\cdot(\log t)^{\frac{5}{2}}. \end{aligned}$$

So

$$\begin{aligned} &r_{1}^{*}=\max \bigl\{ r_{1}(t):t\in[1,e] \bigr\} =k_{1} \biggl(\frac{3k_{1}}{5k} \biggr)^{\frac {3}{2}}-k \biggl( \frac{3k_{1}}{5k} \biggr)^{\frac{5}{2}}=\frac{6\sqrt{15}\cdot k_{1}^{\frac{5}{2}}}{125\cdot k^{\frac{3}{2}}}, \\ &r_{2}^{*}=\max \bigl\{ r_{2}(t):t\in[1,e] \bigr\} =k_{2} \biggl(\frac{3k_{2}}{5k} \biggr)^{\frac {3}{2}}-k \biggl( \frac{3k_{2}}{5k} \biggr)^{\frac{5}{2}}=\frac{6\sqrt{15}\cdot k_{2}^{\frac{5}{2}}}{125\cdot k^{\frac{3}{2}}}. \end{aligned}$$

Take $h_{1}(t)=M_{1}(\log t)^{\frac{3}{2}}, h_{2}(t)=M_{2}(\log t)^{\frac{3}{2}}$, where

$$\begin{aligned} &M_{1}\geq2\mu l_{f}=\frac{24\sqrt{\pi}(18+\sqrt{6})}{53\pi}, \\ &M_{2}\geq2\mu l_{g}=\frac{24\sqrt{\pi}(18+\sqrt{6})}{53\pi}. \end{aligned}$$

Then

$$\begin{aligned} r_{1}(t)={}& \biggl(\frac{8\sqrt{\pi}}{15\pi}+\frac{432\sqrt{6\pi}+144\sqrt {\pi}}{21{,}465}+ \frac{54\sqrt{2\pi}+6\sqrt{3\pi}}{795\pi} \biggr)\cdot (\log t)^{\frac{3}{2}}-\frac{8\sqrt{\pi}}{15\pi}\cdot( \log t)^{\frac {5}{2}} \\ \leq{}& \int_{1}^{e}2\times\frac{12\sqrt{\pi}(18+\sqrt{6})}{53\pi}\cdot(\log t)^{\frac{3}{2}}\frac{ds}{s} \\ ={}& \frac{24\sqrt{\pi}(18+\sqrt{6})}{53\pi}\cdot(\log t)^{\frac {3}{2}} \int_{1}^{e}\frac{ds}{s} \\ \leq{}& M_{1}(\log t)^{\frac{3}{2}}=h_{1}(t), \\ r_{2}(t)={}& \biggl(\frac{8\sqrt{\pi}}{15\pi}+\frac{288\sqrt{2\pi}+1728\sqrt {3\pi}}{21{,}465}+ \frac{12\sqrt{6\pi}+4\sqrt{\pi}}{795\pi} \biggr)\cdot (\log t)^{\frac{3}{2}}-\frac{8\sqrt{\pi}}{15\pi}\cdot( \log t)^{\frac {5}{2}} \\ \leq{}& \int_{1}^{e}2\times\frac{12\sqrt{\pi}(18+\sqrt{6})}{53\pi}\cdot(\log t)^{\frac{3}{2}}\frac{ds}{s} \\ ={}& \frac{24\sqrt{\pi}(18+\sqrt{6})}{53\pi}\cdot(\log t)^{\frac {3}{2}} \int_{1}^{e}\frac{ds}{s} \\ \leq{}& M_{2}(\log t)^{\frac{3}{2}}=h_{2}(t). \end{aligned}$$

In addition,

$$\begin{aligned} f(t,x)&= \biggl(\frac{25\sqrt{15}\cdot k^{\frac{3}{2}}}{18\cdot k_{2}^{\frac{5}{2}}}x+1 \biggr)^{\frac{1}{5}}(k_{2}-k \log t)^{\frac {1}{5}}(\log t)^{\frac{3}{10}} \\ &= \biggl(\frac{25\sqrt{15}\cdot k^{\frac{3}{2}}}{18\cdot k_{2}^{\frac {5}{2}}}x+1 \biggr)^{\frac{1}{5}} \bigl[k_{2}( \log t)^{{\frac{3}{2}}}-k(\log t)^{\frac{5}{2}} \bigr]^{\frac{1}{5}} \\ &= \biggl(\frac{25\sqrt{15}\cdot k^{\frac{3}{2}}}{18\cdot k_{2}^{\frac {5}{2}}}x+1 \biggr)^{\frac{1}{5}} \bigl[r_{2}(t) \bigr]^{\frac{1}{5}} \\ &= \biggl(\frac{25\sqrt{15}\cdot k^{\frac{3}{2}}}{18\cdot k_{2}^{\frac {5}{2}}}xr_{2}(t)+r_{2}(t) \biggr)^{\frac{1}{5}}, \\ g(t,x)&= \biggl(\frac{25\sqrt{15}\cdot k^{\frac{3}{2}}}{18\cdot k_{1}^{\frac{5}{2}}}x+1 \biggr)^{\frac{1}{5}}(k_{1}-k \log t)^{\frac {1}{5}}(\log t)^{\frac{3}{10}} \\ &= \biggl(\frac{25\sqrt{15}\cdot k^{\frac{3}{2}}}{18\cdot k_{1}^{\frac {5}{2}}}x+1 \biggr)^{\frac{1}{5}} \bigl[k_{1}( \log t)^{{\frac{3}{2}}}-k(\log t)^{\frac{5}{2}} \bigr]^{\frac{1}{5}} \\ &= \biggl(\frac{25\sqrt{15}\cdot k^{\frac{3}{2}}}{18\cdot k_{1}^{\frac {5}{2}}}x+1 \biggr)^{\frac{1}{5}} \bigl[r_{1}(t) \bigr]^{\frac{1}{5}} \\ &= \biggl(\frac{25\sqrt{15}\cdot k^{\frac{3}{2}}}{18\cdot k_{1}^{\frac {5}{2}}}xr_{1}(t)+r_{1}(t) \biggr)^{\frac{1}{5}}. \end{aligned}$$

For $\lambda\in(0,1)$, $x\in(-\infty,+\infty)$, $y\in[0,r_{2}^{*}]$,

$$\begin{aligned} &f \bigl(t,\lambda x+(\lambda-1)y \bigr) \\ &\quad= \biggl\{ \frac{25\sqrt{15}k^{{\frac {3}{2}}}}{18k_{2}^{\frac{5}{2}}}r_{2}(t) \bigl[\lambda x+( \lambda-1)y \bigr]+r_{2}(t) \biggr\} ^{\frac{1}{5}} \\ &\quad= \lambda^{\frac{1}{5}} \biggl\{ \frac{25\sqrt{15}k^{{\frac {3}{2}}}}{18k_{2}^{\frac{5}{2}}}r_{2}(t) \biggl[x+ \biggl(1-\frac{1}{\lambda} \biggr)y \biggr]+\frac{1}{\lambda}r_{2}(t) \biggr\} ^{\frac{1}{5}} \\ &\quad = \lambda^{\frac{1}{5}} \biggl\{ \frac{25\sqrt{15}k^{{\frac {3}{2}}}}{18k_{2}^{\frac{5}{2}}}r_{2}(t)x+ \biggl(1-\frac{1}{\lambda} \biggr)\frac {25\sqrt{15}k^{{\frac{3}{2}}}}{ 18k_{2}^{\frac{5}{2}}}r_{2}(t)y+ \frac{1}{\lambda}r_{2}(t) \biggr\} ^{\frac {1}{5}} \\ &\quad \geq \lambda^{\frac{1}{5}} \biggl\{ \frac{25\sqrt{15}k^{{\frac {3}{2}}}}{18k_{2}^{\frac{5}{2}}}r_{2}(t)x+ \biggl(1-\frac{1}{\lambda} \biggr)r_{2}(t)+\frac{1}{\lambda}r_{2}(t) \biggr\} ^{\frac{1}{5}} \\ &\quad = \lambda^{\frac{1}{5}} \biggl\{ \frac{25\sqrt{15}k^{{\frac {3}{2}}}}{18k_{2}^{\frac{5}{2}}}r_{2}(t)x+r_{2}(t) \biggr\} ^{\frac{1}{5}} \\ &\quad = \lambda^{\frac{1}{5}}f(t,x)=\varphi(\lambda)f(t,x), \\ &g \bigl(t,\lambda x+(\lambda-1)y \bigr) \\ &\quad = \biggl\{ \frac{25\sqrt{15}k^{{\frac {3}{2}}}}{18k_{1}^{\frac{5}{2}}}r_{1}(t) \bigl[\lambda x+( \lambda-1)y \bigr]+r_{1}(t) \biggr\} ^{\frac{1}{5}} \\ &\quad= \lambda^{\frac{1}{5}} \biggl\{ \frac{25\sqrt{15}k^{{\frac {3}{2}}}}{18k_{1}^{\frac{5}{2}}}r_{1}(t) \biggl[x+ \biggl(1-\frac{1}{\lambda} \biggr)y \biggr]+\frac{1}{\lambda}r_{1}(t) \biggr\} ^{\frac{1}{5}} \\ &\quad=\lambda^{\frac{1}{5}} \biggl\{ \frac{25\sqrt{15}k^{{\frac {3}{2}}}}{18k_{1}^{\frac{5}{2}}}r_{1}(t)x+ \biggl(1-\frac{1}{\lambda} \biggr)\frac {25\sqrt{15}k^{{\frac{3}{2}}}}{ 18k_{2}^{\frac{5}{2}}}r_{1}(t)y+ \frac{1}{\lambda}r_{1}(t) \biggr\} ^{\frac {1}{5}} \\ &\quad \geq \lambda^{\frac{1}{5}} \biggl\{ \frac{25\sqrt{15}k^{{\frac {3}{2}}}}{18k_{1}^{\frac{5}{2}}}r_{1}(t)x+ \biggl(1-\frac{1}{\lambda} \biggr)r_{1}(t)+\frac{1}{\lambda}r_{1}(t) \biggr\} ^{\frac{1}{5}} \\ &\quad = \lambda^{\frac{1}{5}} \biggl\{ \frac{25\sqrt{15}k^{{\frac {3}{2}}}}{18k_{1}^{\frac{5}{2}}}r_{1}(t)x+r_{1}(t) \biggr\} ^{\frac{1}{5}} \\ &\quad = \lambda^{\frac{1}{5}}g(t,x)=\varphi(\lambda)g(t,x), \end{aligned}$$

here $\varphi(\lambda)=\lambda^{\frac{1}{5}}$. By Theorem 3.1, system (4.1) has a unique solution $(u^{*},v^{*})$ in $\overline{P}_{h,r}$, where

$$\begin{aligned} &r(t)= \bigl(r_{1}(t),r_{2}(t) \bigr) \\ &\phantom{r(t)}= \bigl(k_{1}(\log t)^{\frac{3}{2}}-k(\log t)^{\frac{5}{2}},k_{2}( \log t)^{\frac{3}{2}}-k(\log t)^{\frac{5}{2}} \bigr), \\ &h(t)= \bigl(h_{1}(t),h_{2}(t) \bigr)= \bigl(M_{1}( \log t)^{\frac{3}{2}},M_{2}(\log t)^{\frac {3}{2}} \bigr),\quad t\in[1,e]. \end{aligned}$$

Taking any point $(u_{0},v_{0})\in\overline{P}_{h,r}$, we construct the following sequences:

$$\begin{aligned} &u_{n+1}(t)= \int_{1}^{e}K_{1}(t,s)f \bigl(s,v_{n}(s) \bigr)\frac{ds}{s}+ \int _{1}^{e}H_{1}(t,s)g \bigl(s,u_{n}(s) \bigr)\frac{ds}{s}- \bigl(k_{1}(\log t)^{\frac{3}{2}}-k(\log t)^{\frac{5}{2}} \bigr), \\ &v_{n+1}(t)= \int_{1}^{e}K_{2}(t,s)g \bigl(s,u_{n}(s) \bigr)\frac{ds}{s}+ \int _{1}^{e}H_{2}(t,s)f \bigl(s,v_{n}(s) \bigr)\frac{ds}{s}- \bigl(k_{2}(\log t)^{\frac{3}{2}}-k(\log t)^{\frac{5}{2}} \bigr), \end{aligned}$$

$n=0,1,2,\ldots$ , we have $u_{n+1}(t)\rightarrow u^{*}(t)$, $v_{n+1}(t)\rightarrow v^{*}(t)$ as $n\rightarrow\infty$.
